# FVIII Trafficking Dynamics Across Subcellular Organelles Using CRISPR/Cas9 Specific Gene Knockouts

**DOI:** 10.3390/ijms26136349

**Published:** 2025-07-01

**Authors:** Salime El Hazzouri, Rawya Al-Rifai, Nicole Surges, Melanie Rath, Heike Singer, Johannes Oldenburg, Osman El-Maarri

**Affiliations:** Institute of Experimental Hematology and Transfusion Medicine, University Hospital Bonn, 53127 Bonn, Germany; soulaymahazzouri@gmail.com (S.E.H.); rawya.alrifai@hotmail.com (R.A.-R.); nicole.surges@ukbonn.de (N.S.); melanie.rath@ukbonn.de (M.R.); heike.singer@ukbonn.de (H.S.); johannes.oldenburg@ukbonn.de (J.O.)

**Keywords:** FVIII trafficking, ER/ERGIC chaperones, autophagy-related proteins, CRISPR/Cas9, cell treatment, fluorescence microscopy

## Abstract

Factor VIII (FVIII) interacts with Endoplasmic Reticulum (ER) chaperones Calnexin (CANX) and Calreticulin (CALR) and with ER-Golgi Intermediate Compartment (ERGIC) transporters, Lectin, mannose-binding 1 (LMAN1) and Multiple Coagulation Deficiency 2 (MCFD2). We previously reported that the Gamma-aminobutyric Acid Receptor-associated proteins (GABARAPs) also influence FVIII secretion. Here, we further investigated the intracellular dynamics of FVIII using single and double CRISPR/Cas9 Knockout (KO) models of the abovementioned chaperones as well as the GABARAP proteins in HEK293 cells expressing FVIII. Cellular pathways were manipulated by Brefeldin A (BFA), Chloroquine (CQ), a Rab7 inhibitor, and subjected to glucose starvation. The effect of each KO on FVIII secretion and organelle distribution was assessed by a two-stage chromogenic assay and immunofluorescence (IF) microscopy, prior and upon cell treatments. Using these approaches, we first observed distinct effects of each studied protein on FVIII trafficking. Notably, intracellular localization patterns revealed clustering of FVIII phenotypes in GABARAP^KO^, CANX^KO^, and CALR^KO^ cells together under both basal and treated conditions, an observation that was also reflected in their respective double KO combinations. Besides, a clear involvement of additional components of the endomembrane system was evident, specifically at the *trans*-Golgi space, as marked by FVIII colocalization with the Ras-like proteins in brain (Rab8 and Rab7) and with the Vesicle-Associated Membrane Protein (VAMP8), along with the observed impact of the selected cell treatments on FVIII phenotypes. These outcomes enhance our understanding of the molecular mechanisms regulating FVIII and pave the way for new perspectives, which could be further projected into FVIII replacement, cell and gene therapies.

## 1. Introduction

FVIII is primarily secreted by the liver sinusoidal endothelial cells (LSECs) and to a lesser extent by other extrahepatic sources (e.g., Spleen and lymph nodes) [1,2,3,4,5]. However, LSECs still represent a challenging platform for in vitro cultivation due to factors such as their phenotypic shift in culture, their functional heterogeneity, their low proliferation rate, as well as the necessity of spatial and functional synchronization of these cells with other surrounding liver cells [5]. Current FVIII research is mainly focused on developing treatment and prophylactic regimens for Hemophilia A patients by enhancing the stability and effectiveness of the FVIII protein “exclusively” in the bloodstream [6,7], putting less effort into addressing the basic biosynthesis of FVIII. Despite the advancements made in present therapeutic interventions, our understanding of the early intracellular stages of FVIII processing, before its release into the bloodstream, remains poor and is mostly deduced from knowledge of FVIII expression in artificial systems [8,9,10,11]. Hence, more studies are required to fill this gap, especially in the era of gene and cell therapy.

Upon translocation into the endoplasmic reticulum (ER), monoglycosylated FVIII, holding a Glc_1_Man_9_GlcNAc_2_ N-linked glycan core, undergoes cycles of interactions with chaperones of the protein quality control system such as the Binding Immunoglobulin Protein (BiP), Calnexin (CANX), and Calreticulin (CALR) [11,12]. The recognition of FVIII by ER chaperones CANX and CALR is guided by the activities of ER glucosidases I and II, and glycoprotein glycosyltransferases [8,11,12,13,14]. These interactions lead FVIII into achieving a structural configuration including a high-mannose N-linked glycan core (Man_9_GlcNAc_2_), allowing it to further interact with the ER-Golgi Intermediate Compartment (ERGIC) proteins, the Lectin, mannose-binding 1 (LMAN1), and the Multiple Coagulation Deficiency 2 (MCFD2) proteins, and to be dispatched by the Coat Protein Complex (COPII) vesicles to the Golgi apparatus [12,13]. This step is known to be limited during FVIII biosynthesis and trafficking [9,11,15,16,17]. In fact, due to the tendency of FVIII to aggregate in the ER, only a part of the total synthesized protein is able to leave the ER for forward trafficking [9,17]. Upon escaping the ER, FVIII further travels the ERGIC compartment and progresses through the cisternae of the Golgi apparatus and is cleaved in the *trans*-Golgi by Furin/PACE at residues R1313 and R1648. At this stage, FVIII is ready to be secreted [11,12]. Hereafter, knowledge on *trans*- and post-Golgi downstream trafficking of FVIII remains vague and presents an area that is still overlooked.

Along with these findings, we demonstrate in another study a positive influence of the GABARAP (the gamma aminobutyric acid A receptor) protein on FVIII secretion. We also show that a weak/transient interaction between FVIII and GABARAP could be likely mediated through the B-domain [18]. GABARAP is initially known to function as an adapter protein linking the GABA_A_ receptor to the tubulin of the cytoskeleton (by interacting with the γ2-subunit of the GABA_A_ receptor) thereby influencing inhibitory neurotransmission. GABARAP also belongs to the Atg8 related family of proteins which is highly conserved (yeast to mammals), thereby participating in autophagy-related events [19,20,21,22,23]. Within the Atg8 protein family, GABARAP and its two homologous proteins, GABARAP-Like 1 (GABARAPL1) and GABARAP-Like 2 (GABARAPL2), constitute a distinct sub-family and are primarily involved in the later stages of autophagosome formation and fusion with lysosomes, unlike the Microtubule-associated proteins 1A/1B light chain 3 (LC3s), which function in the earlier initiation and expansion steps of the same process [22,24]. However, due to their likelihood and great sequence homology, it has been difficult to distinguish between the biological functions of the individual GABARAP proteins [22]. This similarity often results in functional redundancy, with overlapping protein interactions and compensatory mechanisms (e.g., the shared characteristic of both GABARAP and GABARAPL1 in binding the γ-subunit of the GABA_A_ receptor, hence taking part in the trafficking and delivery of this receptor to the plasma membrane in the nervous system) [19,22,24].

Interestingly, among all FVIII known and established interacting partners, CALR shares specifically with GABARAP two main characteristics: multifunctionality and multicompartmentality, meaning that both proteins are not restricted to a specific intracellular site or function [25,26,27]. Besides, both GABARAP and CALR were documented to interact with one another, as demonstrated by in vitro assays such as phage display screening, fluorescence titration, surface plasmon resonance (SPR) spectroscopy, as well as cell based assays including either neural or tumorous cells (brain lysate extracts, Neuro-2a and AMO1 cells) [28,29]. Evidence also showed that the deletion of the GABARAP protein diminished the surface exposure of CALR during immunogenic cell death (ICD), subsequently reduced phagocytosis, disrupted the Golgi apparatus, and interrupted the autophagy machinery in multiple myeloma cells [30,31]. However, the exact mechanism by which GABARAP comes together with Calreticulin, as well as the exact location of their interaction, is yet to be explored. So far, these outcomes have been exclusively reported for GABARAP, but not for GABARAPL1 and GABARAPL2.

Based on this background, we aimed to investigate further intracellular aspects of FVIII in HEK293 cells. Our focus included KOs of ER chaperones (CANX and CALR), ERGIC transporter proteins (LMAN1 and MCFD2), and Atg8-related proteins (GABARAP, GABARAPL1, GABARAPL2 and Atg7) in these cells. To achieve this, we first co-stained FVIII with various intracellular markers (proteins) to assess its co-localization within different compartments in the different KO clones, showcasing thereby the imprint of each of the abovementioned proteins on FVIII. We also examined the effects of four cellular treatments including glucose starvation (Glu (-)), Brefeldin A (BFA) and chloroquine (CQ) treatments as well as the inhibition of the GTPase Rab7 by the compound CID 1067700, on FVIII distribution (intracellular) and secretion (extracellular) profiles in these cells. The latter treatments were intended to target specific as well as global cellular trafficking and energetic pathways and to further interfere with their dynamics. As a next step, we generated CRISPR/Cas9 double-KO combinations of FVIII chaperones and GABARAP, in which FVIII secretion, intracellular trafficking, ATP levels, and genetic rescue phenotypes were evaluated.

Our findings revealed a shared influence of GABARAP^KO^, CANX^KO^ and CALR^KO^ on the intracellular distribution of FVIII both under normal and treated conditions. In addition, we identified the specific effects of the generated double gene knockouts on FVIII secretion patterns and noticed similar intracellular phenotypes in the “pairwise” knockouts of CANX, CALR, and GABARAP. We noticed, finally, a clear involvement of the *trans*-Golgi endomembrane system extending beyond the ER and Golgi apparatus in FVIII trafficking.

## 2. Results

### 2.1. FVIII Is Localized to ER, ERGIC, Endosomal, and Proteasomal Compartments

To evaluate the differences in FVIII localization patterns within our cell lines, we aimed to create a standardized pattern of FVIII distribution by looking into wild type cells: HEK28^WT^. To achieve this, we first co-stained FVIII with 21 intracellular proteins using corresponding primary and secondary fluorophore-conjugated antibodies (Table S1).

**Figure 1:**
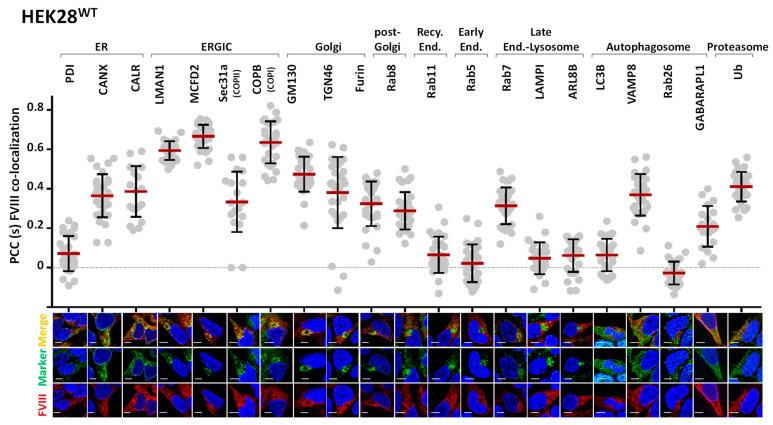
**FVIII is localized to the ER, ERGIC, Endosomal, and Proteasomal Compartments**. *Upper panel*: Dot Plots of Pearson’s Correlation Coefficients (PCC) co-localization values [−1, 1] of FVIII with 21 intracellular markers (Y axis: PCC values [−1, 1], X axis: markers; ER: PDI, CANX, CALR; ERGIC: LMAN1, MCFD2, Sec31a, COPII; Golgi: GM130, TGN46, Furin; Post-Golgi: Rab8; Recycling Endosome: Rab11; Early Endosome: Rab5; Late Endosome: Rab7; Lysosome: LAMPI, ARL8B; Autophagosome; LC3B, VAMP8, Rab26, GABARAPL1; Proteasome: Ubiquitin) in HEK28^WT^. PCCs were extracted using the Zen Blue 2.6 Pro Software. Standard deviation (SD) was calculated using GraphPad Prism 6.07. *Lower panel*: representative immunofluorescence (IF) microscopy merged (automatically) images of FVIII (red) with the different marker proteins (green), respectively. DAPI was used for nuclear staining. Images were acquired using the Axio Observer ApoTome.2 by Carl Zeiss at 40× magnification with an oil immersion objective (numerical aperture 1.4), providing an optical resolution of approximately 240 nm in X and Y axes. A 702 monochrome camera with a 6.5 µm pixel pitch was used, yielding a pixel-based resolution of approximately 147 nm. Scale bar 5 µm.

As expected, FVIII was observed to co-localize with its known interacting partners: the ER chaperones CANX (PCC = 0.36, SD = 0.11) and CALR (PCC = 0.39, SD = 0.13), as well as the two components of the cargo receptor complex LMAN1 (PCC = 0.59, SD = 0.05) and MCFD2 (PCC = 0.67, SD = 0.06) in the ERGIC compartment. FVIII was consistently present with markers of the COPII and COPI vesicles (Sec31a PCC = 0.33, SD = 0.15 and COPB PCC = 0.64, SD = 0.10), and proteins of the *cis-* and *trans*-Golgi networks (Golgi-matrix protein 130 (GM130) PCC = 0.48, SD = 0.09 and *trans*-Golgi network protein 46 (TGN46) PCC = 0.38, SD = 0.18). These findings were expected, given that FVIII follows the ER to Golgi secretory route upon synthesis [11,12]. Moreover, moderate co-localizations of FVIII with the Ras-like proteins in brain Rab8 (PCC = 0.3, SD = 0.09); Rab7 (PCC = 0.31, SD = 0.09); and the Vesicle Associated Membrane Protein VAMP8 (PCC = 0.37, SD = 0.1), markers of post-Golgi secretory vesicles, late endosomes, and autophagosomes, respectively, were observed. Our data did not show a significant presence of FVIII in lysosomal-related vesicles (LAMP1 PCC = 0.05, SD = 0.08; ARL8B PCC = 0.06, SD = 0.08), nor in early (Rab5 PCC = 0.02, SD = 0.09) or recycling endosomal (Rab11 PCC = 0.07, SD = 0.09) compartments. Although FVIII showed moderate co-localization with VAMP8, a marker involved in the direct control of membrane fusion between autophagosomal and lysosomal structures, it did not co-localize to other autophagosome-related markers, such as LC3B (PCC = 0.06, SD = 0.08) and Rab26 (PCC = −0.03, SD = 0.06) (Figure 1).

### 2.2. FVIII Co-Localization with Intracellular Markers Places GABARAP^KO^, CANX^KO^, and CALR^KO^ Clones in the Same Cluster

**Figure 2:**
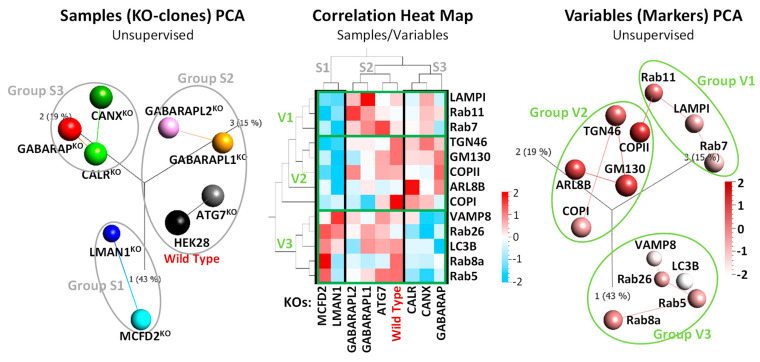
**FVIII Co-localization with Intracellular Markers places GABARAP^KO^, CANX^KO^, and CALR^KO^ Clones in the Same Cluster**. Multi-angled visualization (unsupervised 3D-Principal Component Analysis (PCA) plots and Heat Map matrix generated using the Qlucore Omics Explorer 3.6) of average FVIII co-localization values (PCCs) with 13 intracellular markers (proteins) in eight CRISPR/Cas9-KOs and HEK28^WT^. *Left*, 3D-PCA plot of sample (cell lines) and *right*, of variables (intracellular markers) clustering. *Middle*, correlation Heat Map visualization combining both samples’ and variables’ PCA plots. The color scale ranges from −2 (blue, negative correlations) to 2 (red, positive correlations). Values close to 0 (white) indicate no correlation. Each square in the Heat Map represents a PCC score.

We went forward to analyze FVIII patterns within the eight single CRISPR/Cas9-KO clones (CANX^KO^, CALR^KO^, LMAN1^KO^, MCFD2^KO^, GABARAP^KO^, GABARAPL1^KO^, GABARAPL2^KO^ and Atg7^KO^). The average Pearson’s correlation/co-localization coefficients (PCC) were integrated into tabular formats, plotting cell lines (samples) against markers (variables), and then utilized for the generation of principal component analysis (PCA) plots and Heat Map Matrices (Figure 2). In this section, we reduced the number of intracellular markers tested in order to avoid biases arising from potentially imputed values in the KO clones.

Hierarchical clustering revealed three sample arrays, S1: consisting of LMAN1^KO^ and MCFD2^KO^ (ERGIC transporters related-KOs) along PC1 (43%); S2: including GABARAPL1^KO^, GABARAPL2^KO^ along PC3 (15%), and ATG7^KO^ (Atg8 related-KOs) and HEK28^WT^ between PC1 (43%) and PC3 (15%); and S3: comprising CALR^KO^, CANX^KO^ (ER-chaperones-KOs), with, intriguingly, GABARAP^KO^, along PC2 (19%) (Figure 2, left PCA plot and middle Heat Map). Moreover, clustering of variables divided the various intracellular markers into three main groups, namely, V1: including LAMP1, Rab11, and Rab7 (lysosomal, recycling and late endosomal); V2: consisting of TGN46, GM130, COPII, ARL8B, and COPI (ERGIC and classical secretory route markers); and V3, containing VAMP8, Rab26, LC3B, Rab8a, and Rab5 (autophagosomal, early endosomal, and post-Golgi markers) (Figure 2, right PCA plot and middle Heat Map).

The obtained results not only confirm the anticipated sample clustering but also aligned with established information on FVIII assisting ER chaperones and ERGIC transporter proteins, where, specifically, CANX^KO^ and CALR^KO^ closely associated in S3, while LMAN1^KO^ and MCFD2^KO^ form together S1, as expected. Interestingly, GABARAP^KO^ was part of cluster S1 alongside CANX^KO^ and CALR^KO^ rather than with its homologous protein knockouts, GABARAPL1^KO^ and GABARAPL2^KO^, which were grouped with Atg7^KO^ in cluster S2. The latter reinforces the similar impact of GABARAPL1 and GABARAPL2 on the FVIII machinery, and the functional divergence of GABARAP, which is in more proximity to CANX and CALR.

It is also essential to highlight the striking contrast observed in the co-localization of FVIII with intracellular markers between S1 (LMAN1^KO^ and MCFD2^KO^) and S3 (CALR^KO^, CANX^KO^, and GABARAP^KO^). Specifically, S1 showed relatively lower co-localization with V1 and V2 markers, whereas S3 exhibited higher co-localization with the same markers. Conversely, V3 markers exhibited higher co-localization with FVIII in the S1 group and lower co-localization in the S3 group (Figure 2, middle Heat Map).

### 2.3. Cell Treatments Align GABARAP^KO^ with CALR^KO^ in FVIII Secretion and Additionally Highlight the Contribution of the Endomembrane System in FVIII Trafficking

**Figure 3:**
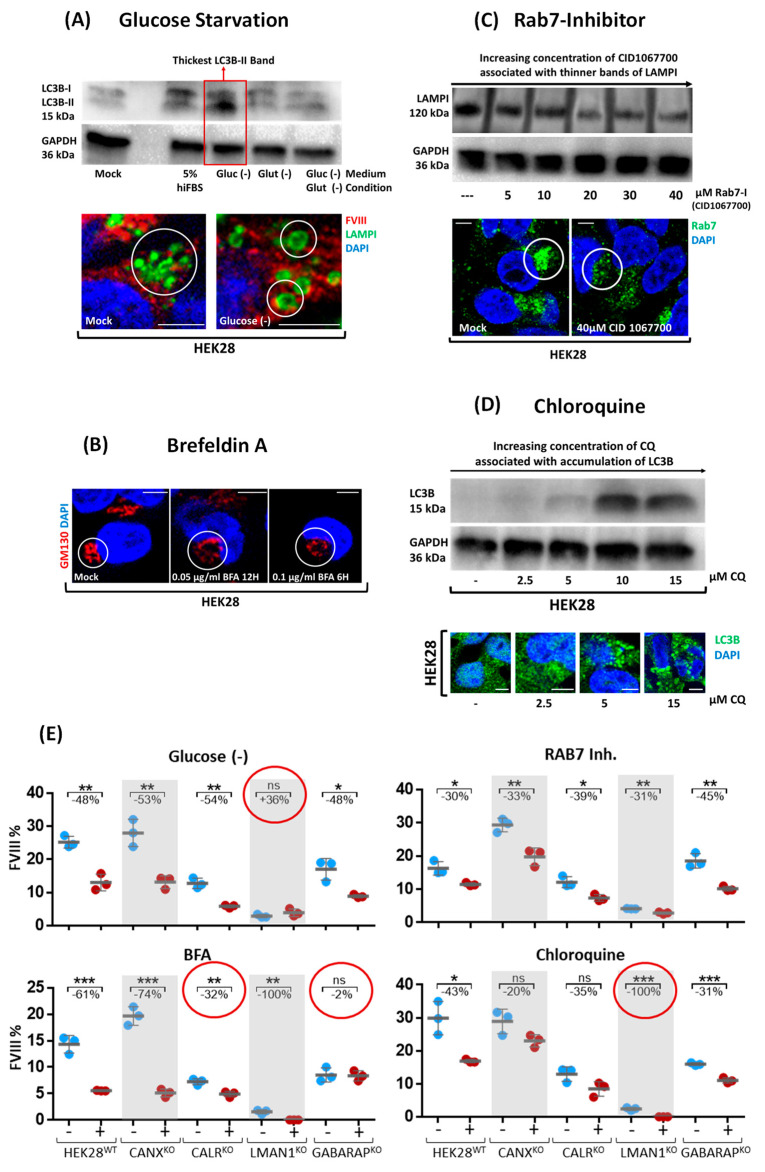
**Cell Treatments align GABARAP^KO^ with CALR^KO^ in FVIII secretion and additionally highlight the contribution of the endomembrane system in FVIII trafficking.** (**A**) Glucose Starvation (Glu (-)) was established by detecting autophagy stimulation, selected based on the thickest detected LC3B-II band in Western Blot (WB). A clear variation in the size of lysosomes (bigger vesicles marked by LAMPI in green) was also observed. (**B**) Different Brefeldin A (BFA) concentrations and time points were tested revealing a disruption of the Golgi morphology marked by the *cis*-Golgi marker GM130 (red). (**C**) Rab7 Inhibition was accomplished by applying 40 µM CID1067700 upon detecting thinner bands of LAMPI with increasing concentrations (5 µM to 40 µM) of CID1067700. (**D**) Increasing concentrations of chloroquine (CQ) (2.5 µM to 15 µM) reflected accumulated LC3B. Thicker LC3B bands were also detected by Western Blot (WB) and IF staining (green). Images were acquired using the ApoTome.2 microscope by Carl Zeiss at 40× magnification with an oil immersion objective (numerical aperture 1.4), providing an optical resolution of approximately 240 nm in x and y axes. A 702 monochrome camera with a 6.5 µm pixel pitch was used, yielding a pixel based resolution of approximately 147 nm. Scale Bar for microscopy images is 5 µm. (**E**) FVIII secretion percentages obtained by two-stage-chromogenic assay (Y axis) in HEK28^WT^ and five CRISPR/Cas9-KO cells plotted on the X axis, treated (red dots) with glucose starvation, Brefeldin A, Rab7 inhibition (CID 1067700), Chloroquine (CQ), and mock conditions (blue dots) (n.s.: non significant; *: *p* < 0.05; **: *p* < 0.01; ***: *p* < 0.001).

Cellular treatments were administered to disrupt specific compartments and vesicles in HEK28^WT^, CANX^KO^, CALR^KO^, LMAN1^KO^, and GABARAP^KO^ cells. The aim is to evaluate the impact of each treatment on FVIII secretion and intracellular localization. A thick LC3B-II band was observed upon glucose starvation of HEK28^WT^, indicating an activation of the autophagy machinery marked by a higher presence of the lipidated form of the LC3B protein; in parallel, we perceived, through IF visualizations, an enlargement of lysosomal vesicles following the labeling of the LAMPI protein in green (Figure 3A). After subjecting single KO clones to glucose starvation (Glu (-)), a statistically significant decrease in FVIII accumulation in the media (when compared to untreated mock) was similarly observed in HEK28^WT^ (48%; *p* < 0.01), CANX^KO^ (53%; *p* < 0.01), CALR^KO^ (54%; *p* < 0.05), and GABARAP^KO^ (48%; *p* < 0.05), whereas LMAN1^KO^ exhibited a non statistically significant opposite effect of very small magnitude, resulting in a 36% increase in FVIII secretion (Figure 3E, upper panel left Dot Plots).

Next, we treated the cells with Brefeldin A (BFA), a fungal compound that is well characterized as an antiviral antibiotic [32]. BFA inhibits protein secretion by interrupting the ER-to-Golgi secretory flow [32]. Trials were initiated in HEK28^WT^ with concentrations ranging from 1–10 µg/mL BFA at time intervals of: 0.5, 1, 2, 4, 6, and 12. We have observed that a time interval of 30 min was already sufficient to detect a disrupted Golgi morphology accompanied by a retention/retraction of the Golgi apparatus in the endoplasmic reticulum membranes, under incubation with all three tested concentrations. However, at least 12–16 h were needed to detect a slight accumulation of FVIII in the media. Accordingly, BFA concentrations were decreased to a range of 0.05–0.1 µg/mL over durations of 6, 12, and 20 h. The latter conditions showed the expected modifications in Golgi morphology and its organization (Figure 3B). We subsequently carried out an incubation of the KO cells with 0.05 µg/mL BFA for 20 h. Our results showed a statistically significant but variable decrease in FVIII secretion across the different cell lines, compared to untreated, in HEK28^WT^ (61%; *p* < 0.001), CANX^KO^ (74%; *p* < 0.001), and CALR^KO^ (32%; *p* < 0.01) cells. However, BFA completely halted FVIII secretion in LMAN1^KO^ (0%; *p* < 0.01). Remarkably, the GABARAP^KO^ exhibited minimal impact from BFA treatment, with only a 2% non significant decrease, suggesting that BFA acts in a GABARAP dependent manner, as its effect becomes negligible in the absence of the GABARAP protein (Figure 3E, lower panel left Dot Plot).

We next inhibited Rab7, a GTPase protein within the Ras superfamily recognized for its involvement in lysosomal biogenesis and fusion with late endocytic structures. Inhibiting Rab7 is expected to diminish this process, resulting in a decreased density of lysosomal vesicles [33]. To assess whether this inhibition affects FVIII trafficking and secretion mechanisms, we treated the cells with CID1067700, a compound known to competitively inhibit Rab7 [33]. Gradually increasing concentrations of CID1067700 (5 µM, 10 µM, 20 µM, 30 µM, and 40 µM) were administered, and cells were allowed to incubate for 24 to 36 h. The correlation between increasing CID1067700 concentrations and a reduction in the size of the LAMPI band was observed by Western Blot (WB) (Figure 3C), signifying treatment success and efficiency. Cells were then exposed to 40 µM of CID1067700, given the absence of detected cell toxicity. Reduced FVIII activity was noted across all cell lines: HEK28^WT^ (30%; *p* < 0.05), CANX^KO^ (33%; *p* < 0.01), CALR^KO^ (39%; *p* < 0.05), LMAN1^KO^ (31%; *p* < 0.01), and GABARAP^KO^ (45%; *p* < 0.01), with CALR^KO^ and GABARAP^KO^ being the most affected by the inhibition of the Rab7 protein (Figure 3E, upper panel right Dot Plot).

Lastly, increasing concentrations of CQ (2.5–15 µM) caused accumulation of the LC3B protein. This was detected microscopically as more LC3B compact vesicular dots and by WB as a gradually appearing LC3B thicker band (Figure 3D). This was expected since CQ is known to inhibit the autophagic flux by altering lysosomal enzyme activity and pH levels, thereby blocking fusion with autophagosomes [34]. Upon incubation with 10 µM CQ, we have noticed a decrease in FVIII secretion (FVIII: AC) across all cell lines. This reduction varied among the different KOs, with a 43% decrease in HEK28^WT^ (*p* < 0.05), 20% in CANX^KO^ (ns), and similar decreases of 35% (ns) and 31% (*p* < 0.001) in CALR^KO^ and GABARAP^KO^, respectively. Residual FVIII activity was observed in all cells except in LMAN1^KO^, where secretion was completely abolished (*p* < 0.001); a similar result which was observed when this KO was subjected to BFA treatment (Figure 3E, lower panel right Dot Plot).

### 2.4. Co-Localization of FVIII with Key Intracellular Landmarks Following Cell Treatments Positions GABARAP^KO^ Close to CANX^KO^

**Figure 4:**
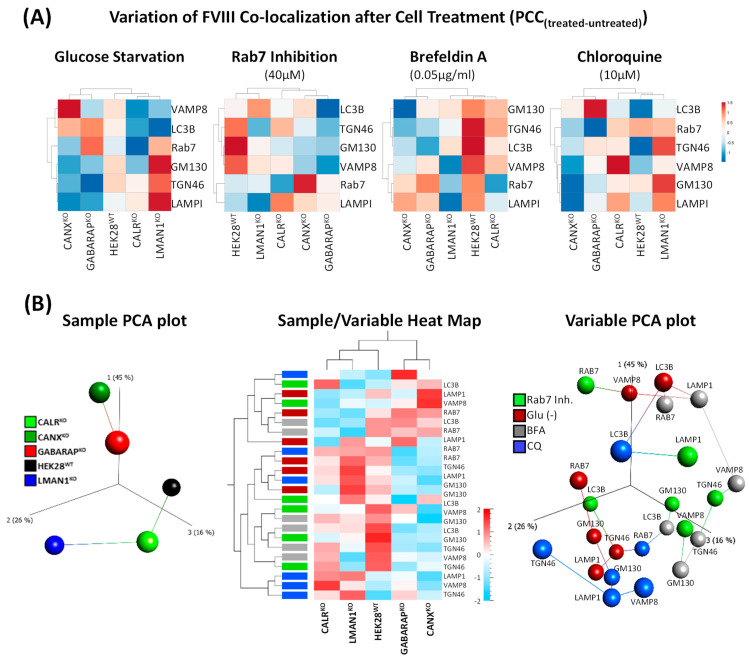
**Co-localization of FVIII with key intracellular landmarks following cell treatments positions GABARAP^KO^ close to CANX^KO^.** (**A**) Heat Maps representing clustering of knockout cell lines (HEK28^WT^, CANX^KO^, CALR^KO^, LMAN1^KO^, and GABARAP^KO^) based on PCC values reflecting FVIII co-localization with six intracellular markers (GM130, TGN46, Rab7, VAMP8, LAMPI and LC3B) in individual treatment conditions. PCC values were computed as PCC_treat._—PCC_untreat._, for each treatment. Treatments include glucose starvation, Rab7 inhibition (40 µM), BFA (0.05 µg/mL) and CQ (10 µM). Heat Maps Color code ranges from −1.5 (blue-negative correlations) to 1.5 (red-positive correlations). Values close to 0 (white) indicate no correlation. Each square represents a PCC_treat-untreat._ value. (**B**) Multiangled global (combined treatments data) visualization (upon subtraction of PCC_treat.-_ PCC_untreat._ values) using 3D unsupervised PCA plots and a Heat Map Matrix showing clustering of samples (left) and variables (right) and a combination of both in the middle Heat Map matrix. The color scale in the middle Heat Map ranges from −2 (blue, negative correlations) to 2 (red, positive correlations). Values close to 0 (white) indicate no correlation. Each square represents a PCC_treat-untreat._ value.

After demonstrating the effects of the four selected treatments on FVIII secretion in individual KO clones (Figure 3), we aimed to link this phenotype to the localization of FVIII with key intracellular markers. To this end, only six proteins were selected: GM130 (*cis-*Golgi network), TGN46 (*trans*-Golgi network); Rab7 (late endosomes-lysosomes); LAMPI (lysosomes), VAMP8 (endosomal and autophagosomal structures), and LC3B (autophagosomes). This choice was made to detect changes at key landmarks, including the Golgi apparatus, the endosomes, the lysosomes, and the autophagosomes (Figure 4). To highlight significant differences in mock samples across the different cell lines, PCC values of FVIII co-localization with the chosen six markers obtained upon cell treatment were deduced from values of FVIII co-localization with the same markers before cell treatment conditions (Figure 4A).

Based on this approach, four individual Heat Maps were generated to visualize FVIII across various organelles under the four treatment conditions (glucose starvation, Rab7 inhibition, BFA, and CQ). To assess the specific effect of individual treatments, we first monitored HEK28^WT^. In HEK28^WT^, glucose starvation had minimal impact on FVIII trafficking, as evidenced by the lack of significant differences in co-localization across all organelles. In contrast, Rab7 inhibition disrupted FVIII trafficking, particularly affecting its localization to late endosomes (Rab7) and lysosomes (LAMP1). Furthermore, BFA resulted in reduced FVIII secretion (Figure 3) and increased intracellular accumulation, indicating that FVIII was retained intracellularly upon blockage of the ER-Golgi dependent route. CQ, consistent with its known mechanism of action, impaired trafficking at the *trans*-Golgi network (TGN46), autophagosomes (LC3B), and lysosomes (LAMPI), altering FVIII localization accordingly. (Figure 4A).

Furthermore, each KO cell line exhibited distinct alterations in FVIII localization in response to the different treatments. However, strikingly, GABARAP^KO^ and CANX^KO^ clones consistently demonstrated highly similar profiles across all treatment. This strong similarity suggests a potential functional convergence between the GABARAP and CANX pathways in regulating FVIII trafficking under conditions of cellular stress (Figure 4A).

Supporting this observation, global PCA and Heat Map clustering of data combining all treatment conditions (Figure 4B) revealed that GABARAP^KO^ and CANX^KO^ cells clustered closely together, once more, reinforcing the hypothesis of a shared or overlapping roles in FVIII processing and organellar dynamics. Interestingly, the global PCA plot further revealed that markers affected by glucose starvation and CQ treatments (represented by red and blue spheres, respectively) formed a distinct cluster, while those influenced by Rab7 inhibition and BFA treatments (green and grey spheres, respectively) predominantly grouped together, indicating treatment-specific trafficking patterns (Figure 4B).

**Figure 5:**
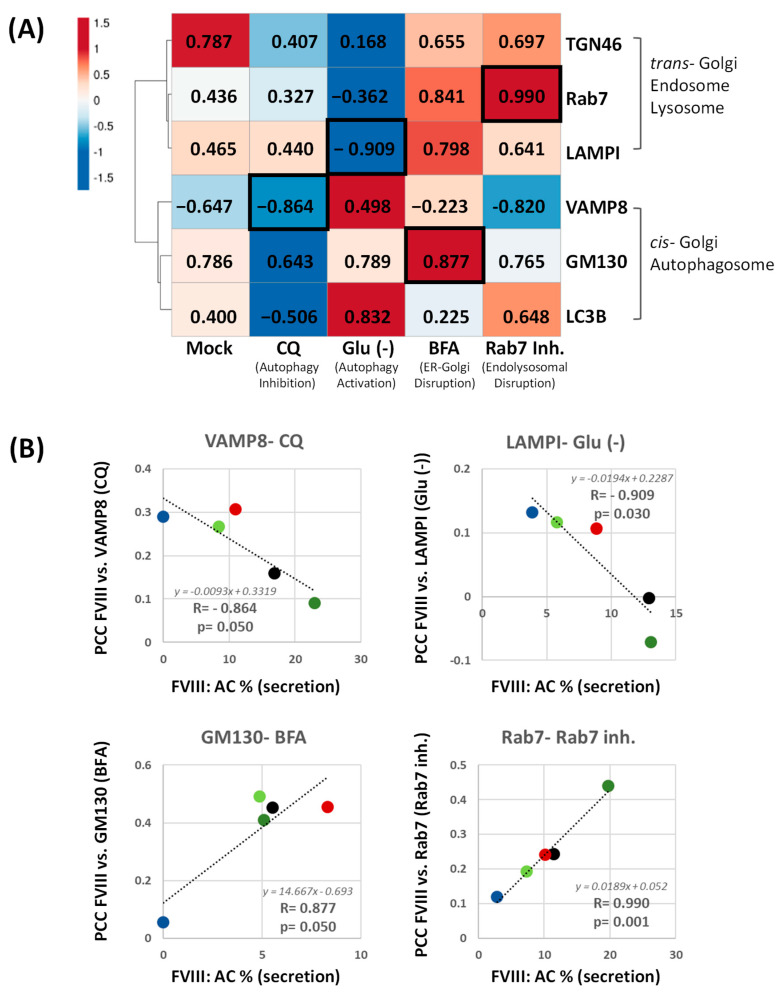
**Correlation Analysis of FVIII Secretion vs. FVIII Intracellular Co-localization Before and After Cell Treatment.** (**A**) Heat Map showing correlation values (Pearson’s R, range [−1 to 1]) between FVIII secretion (% Activity) and FVIII intracellular co-localization (PCC values) with intracellular proteins (GM130, TGN46, Rab7, VAMP8, LAMP1, and LC3B) in different treatment conditions. Correlation Clustering was applied to the intracellular proteins. Conditions include mock (untreated) and treatments with chloroquine (CQ), glucose starvation (Glu (-)), Brefeldin A (BFA), and Rab7 inhibition (Rab7 inh.). Black square contours represent significant correlations. (**B**) Scatter plots displaying correlations (Pearson’s R, range [−1 to 1]) between FVIII secretion (X axis; FVIII secretion levels) and FVIII co-localization (Y axis; PCC values) at VAMP8 (CQ), LAMPI (Glu (-)), GM130 (BFA) and Rab7 (Rab7 inh.). Colored dots represent the different cell lines HEK28^WT^ (black), CANX^KO^ (dark green), CALR^KO^ (light green), LMAN1^KO^ (blue) and GABARAP^KO^ (red).

Correlation analysis under basal untreated (mock) conditions demonstrated that FVIII secretion positively correlated with its trafficking to *cis-* and *trans*-Golgi networks (R_GM130_ = 0.786; R_TGN46_ = 0.787); Rab7 (R_Rab7_ = 0.436), LAMP1 (R_LAMPI_ = 0.465), and LC3B (R_LC3B_ = 0.400), yet negatively with VAMP8 (R_VAMP8_ = –0.647). CQ weakened these associations, particularly diminishing FVIII’s correlation with Golgi (R_GM130_ = 0.643, R_TGN46_ = 0.407), late endosomal (R_Rab7_ = 0.327) and autophagosomal markers (R_VAMP8_ = −0.864; R_LC3B_ = −0.503), reflecting impaired secretory trafficking. Conversely, glucose starvation enhanced FVIII association with autophagic markers (R_VAMP8_ = 0.498 and R_LC3B_ = 0.823) while reducing co-localization with *trans*-Golgi (R_TGN46_ = 0.168) and endosomal markers (R_Rab7_ = −0.362; R_LAMPI_ = −0.909), suggesting a shift toward unconventional, autophagy-related secretion pathways under metabolic stress. Under both BFA and Rab7 inhibition, FVIII secretion was more correlated with GM130 (R_GM130_ = 0.877) and Rab7 (R_Rab7_ = 0.990), respectively, the exact locations where both compounds interfere, indicating that reduced secretion under these conditions was associated with disrupted trafficking of FVIII in the early (ER to Golgi) secretory pathway and *trans*-Golgi sorting pathway, respectively (Figure 5).

### 2.5. Double Knockouts of ER/ERGIC Chaperones and GABARAP Reveal Distinct Effects on FVIII Secretion and Trafficking

**Figure 6:**
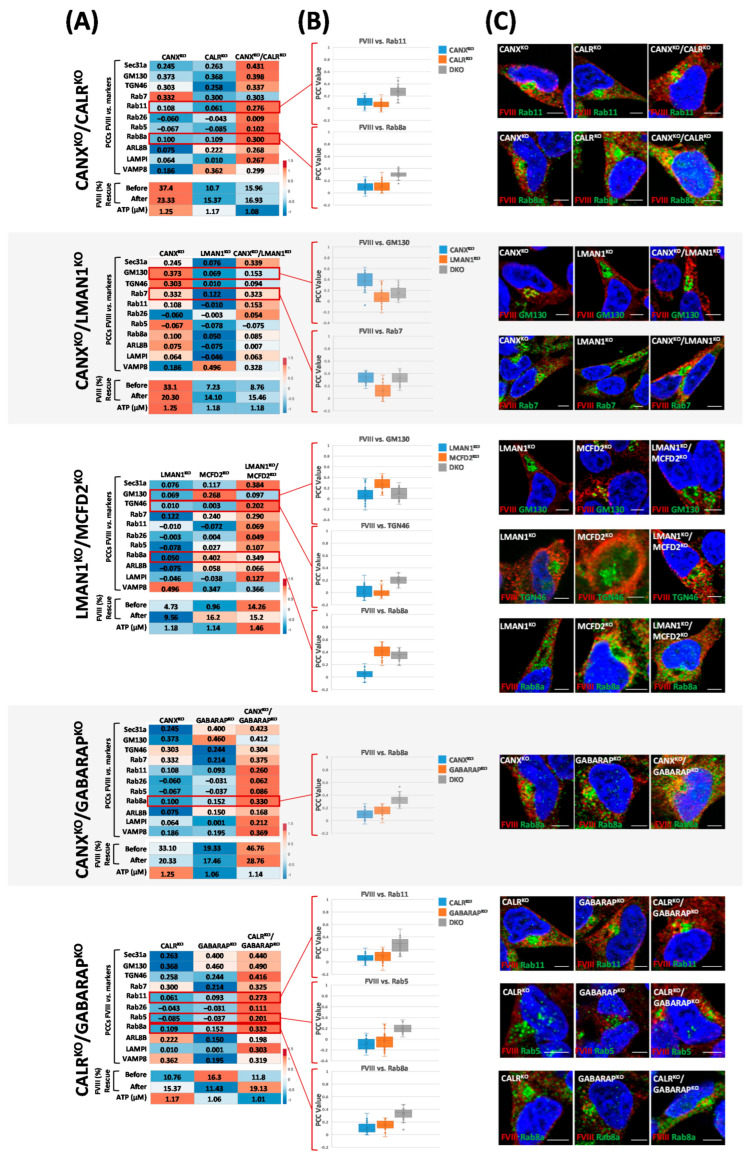
**Double knockouts of ER/ERGIC chaperones and GABARAP reveal distinct effects on FVIII secretion and trafficking.** (**A**) Correlation Heat Maps of (1) FVIII co-localization with intracellular markers (Sec31a, GM130, TGN46, Rab5, Rab7, Rab8a, Rab11, Rab26, ARL8B, LAMP1, and VAMP8), quantified using Pearson Correlation Coefficient (PCC) values [−1; 1] extracted by the Zen Blue 2.6 Pro Software, (2) measurements of FVIII secretion (residual FVIII activity in the media) assessed via a two-stage chromogenic assay before and after genetic rescue (%), (3) as well as residual ATP concentrations (µM) determined using a luciferase-based assay. (**B**) Boxplots comparing PCC values (Y axis: [−1, 1]) between single knockouts (SKOs) and their corresponding double knockouts (DKOs), highlighting markers with the most pronounced differences. (**C**) Representative microscopy images of intracellular markers with the most significant changes between double-KOs (DKOs) and their respective single-KOs (SKOs). FVIII is shown in red, intracellular markers in green, and nuclei (DAPI staining) in blue. Images were acquired using the Axio Observer ApoTome.2 by Carl Zeiss microscope at 40× magnification with an oil immersion objective (numerical aperture 1.4), providing an optical resolution of approximately 240 nm in x and y axes. A 702 monochrome camera with a 6.5 µm pixel pitch was used, yielding a pixel based resolution of approximately 147 nm. Scale bar: 5 µM.

Following the generation and characterization of double CRISPR/Cas9-KOs targeting FVIII assisting chaperones—CANX, CALR, LMAN1, and MCFD2—as well as GABARAP (Figure S1), FVIII secretion levels (activity in the media) were assessed using a two-stage chromogenic assay. Consistent with our earlier results, CANX^KO^ increased FVIII secretion, while CALR^KO^ reduced it in comparison to HEK28^WT^ (Figure 3E). When combined, CANX^KO^/CALR^KO^ showed a secretion value between both single-KOs, suggesting an additive effect that converged to yield a specific FVIII secretion level. Furthermore, intracellular tracking of FVIII in CANX^KO^/CALR^KO^ showed significant differences compared to the respective single CANX^KO^ and CALR^KO^, particularly with endocytic and exocytic vesicle markers Rab11 and Rab8 (Figure 6, First panel). Subsequently, when both ER membrane proteins CANX and LMAN1 were simultaneously absent, FVIII secretion was predominantly influenced by the absence of LMAN1, showing a marked reduction closer to the one observed upon single LMAN1^KO^. This outcome was accompanied by a diminished FVIII presence at the Golgi (GM130 and TGN46), consistent with the critical role of LMAN1 in ER-Golgi transport. Besides, a skew toward the CANX^KO^ phenotype with Rab7 (late endosomes) and the LMAN1^KO^ phenotype with VAMP8 (autophagosomes) was apparent (Figure 6, second panel). Furthermore, both single LMAN1^KO^ and MCFD2^KO^ reduced FVIII secretion and impaired FVIII co-localization with Sec31a (COPII vesicles), TGN46 (Golgi), and Rab7 (late endosomes). However, LMAN1^KO^/MCFD2^KO^ presented a distinct phenotype. Despite reduced secretion, FVIII levels in the DKO remained higher than those observed in either of the respective SKOs. Interestingly, co-localization patterns were scrambled where FVIII presence with COPII vesicles (Sec31a) was higher in the DKO, while GM130-associated localization resembled the LMAN1^KO^. Co-localization with TGN46 was more pronounced in the DKO, and Rab8 association was relatively high and skewed toward the MCFD2^KO^ phenotype (Figure 6, third panel). As for GABARAP and the ER chaperones, remarkably, the secretion profiles in both DKOs tended to mirror the effects of either CALR^KO^ or CANX^KO^, suggesting that the loss of either ER chaperone overrode GABARAP’s influence on FVIII secretion. The synergistic effect of CANX^KO^/GABARAP^KO^ was paralleled by increased co-localization of FVIII with Rab11 and Rab8 vesicle markers compared to the respective CANX^KO^ and GABARAP^KO^. For CALR^KO^/GABARAP^KO^, despite reduced FVIII secretion, FVIII associated more with Rab11, Rab5, and Rab8 (Figure 6, fourth and fifth panels).

Higher ATP levels were observed in the single CANX^KO^ (1.25 µM), whereas they were lower in CALR^KO^ (1.17 µM), LMAN1^KO^ (1.18 µM), CANX^KO^/CALR^KO^ (1.08 µM), and CANX^KO^/LMAN1^KO^ (1.18 µM). Additionally, LMAN1^KO^/MCFD2^KO^ exhibited higher ATP levels (1.46 µM) compared to the corresponding individual LMAN1^KO^ (1.18 µM) and MCFD2^KO^ (1.14 µM). These outcomes aligned with trends of FVIII secretion levels suggesting a clear influence of cellular energetic status on FVIII secretion efficiency, particularly in ER chaperone- and trafficking-related KOs. Nevertheless, in the pairwise DKOs involving ER chaperones and GABARAP, no consistent correlation was observed between ATP levels and FVIII secretion levels, suggesting that while cellular ATP levels may reflect the secretory capacity under certain conditions, they are not the sole determinant of FVIII trafficking and secretion (Figure 6A).

**Figure 7:**
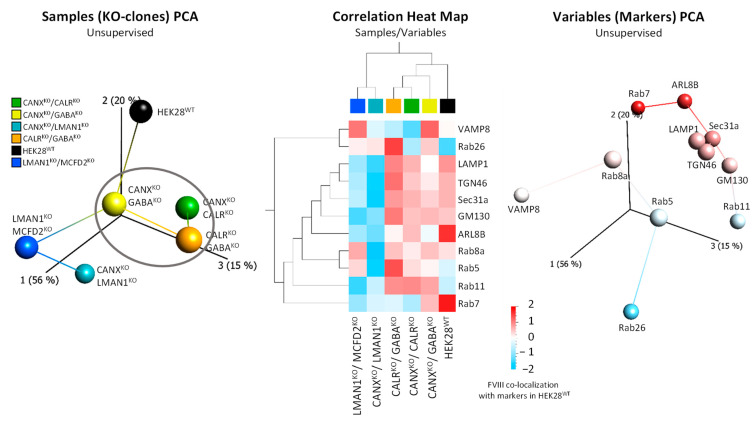
**Analysis of FVIII Co-localization with 11 Intracellular Markers place GABARAP, CANX and CALR DKO combinations in The Same Cluster.** Multiangled visualization (unsupervised 3D-Principal Component Analysis (PCA) plots and Heat Map matrix) of average FVIII co-localization values (PCCs) with 11 intracellular markers in five CRISPR/Cas9 double knockout combinations and HEK28^WT^ (designated by a color code left). Left, 3D-PCA plot of sample (cell lines) and right, of variables (intracellular markers) clustering. Middle, Heat Map visualization combining both samples’ and variables’ PCA plots. The color scale ranges from −2 (blue, negative correlations) to 2 (red, positive correlations). Values close to 0 (white) indicate no correlation. Each square in the Heat Map represents a PCC score.

Finally, PCA plots of FVIII co-localization values with 11 intracellular markers in the DKO combinations showed, interestingly that like CANX^KO^, CALR^KO^ and GABARAP^KO^ clustered together, slightly further away from the cluster containing LMAN1^KO^ and MCFD2^KO^ (Figure 2), DKO combinations involving pairs of deletions of CANX, GABARAP, and CALR clustered together away from the cluster containing CANX^KO^/LMAN1^KO^ and LMAN1^KO^/MCFD2^KO^ combinations (Figure 7).

## 3. Discussion

**Figure 8:**
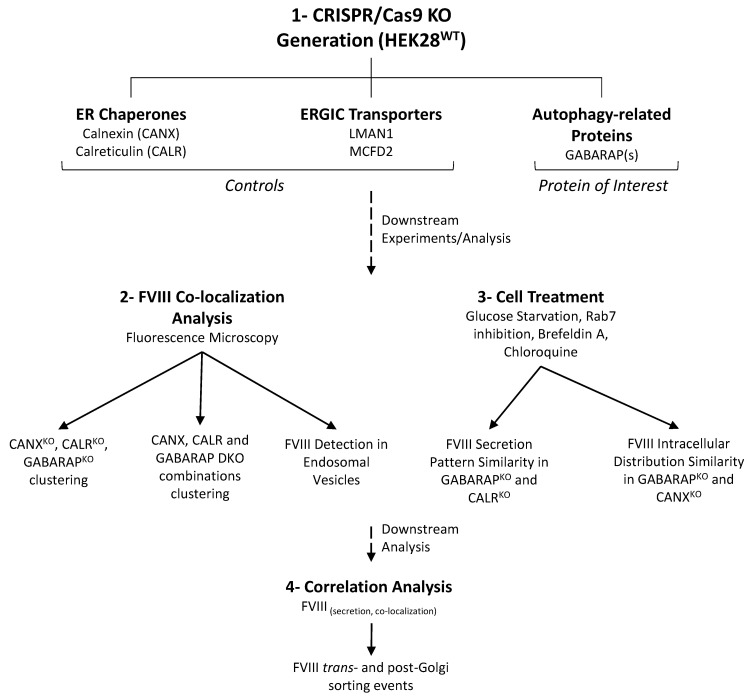
**Schematic representation of the main experimental strategy and findings**. (1) Single and double CRISPR/Cas9 knockouts were generated using a HEK293 clone stably expressing FVIII (HEK28^WT^). Targeted genes included CANX and CALR (ER chaperones FVIII interactors), LMAN1 and MCFD2 (ERGIC FVIII transporters), used as controls, and GABARAP(s) (Autophagy-related protein of interest). (2) Cell lines were validated and subjected to fluorescence microscopy by FVIII co-staining with intracellular markers and to (3) specific cell treatments. Clustering analysis based on FVIII intracellular distribution grouped CANX^KO^, CALR^KO^, and GABARAP^KO^, along with their respective double KOs in one cluster. Upon chemical treatment, GABARAP^KO^ showed secretion profiles resembling CALR^KO^ and intracellular FVIII distribution similar to CANX^KO^. (4) Further correlation analysis between FVIII secretion and co-localization patterns indicated a role for *trans*- and post-Golgi sorting in FVIII trafficking.

The native dynamics of FVIII biosynthesis, trafficking, and secretion have been challenging to identify due to the absence of a robust LSEC model (the main FVIII-secreting cells), which in turn impaired rigorous in vitro culture studies involving FVIII [1,2,3]. In this study, we used a stable HEK293 FVIII expressing system (HEK28^WT^) to explore beyond what we know about the intracellular life of FVIII. Here, we investigated the effects of CRISPR/Cas9-KOs of ER chaperones CANX and CALR, ER-to-Golgi FVIII transport proteins LMAN1 and MCFD2 (as controls), and of GABARAP proteins on FVIII biogenesis (a schematic flow of the executed work is presented in Figure 8).

LMAN1^KO^ and MCFD2^KO^ presented close phenotypes showing clear disruptions of FVIII secretion and trafficking in the ERGIC, Golgi, and further endosomal compartments (Figure 2 and Figure 6). This phenotype was also paralleled in LMAN1^KO^/MCFD2^KO^ double-deficient cells; however, in this combination, neither FVIII secretion nor its shuttle to the Golgi apparatus was as drastically affected as in the respective single LMAN1^KO^ and MCFD2^KO^. A potential explanation of this outcome could be the presence of a positive epistatic effect upon complete deletion of the cargo receptor. This could result in an activation of compensatory mechanisms (involving other proteins facilitating FVIII transport (e.g., calcium calcium-dependent L-type lectins like ERGL, VIP36, VIPL, etc.), or in an increase in bulk flow trafficking as a cellular response to maintain energy homeostasis and cellular equilibrium [35,36]. Supporting this hypothesis, residual ATP (cellular energetic levels) and FVIII secretion levels were higher in the DKO compared to either of the respective SKOs (Figure 6).

Furthermore, GABARAP^KO^ aligned closely to CANX^KO^ and CALR^KO^ under basal and treatment conditions (Figure 2, Figure 3 and Figure 4), yet GABARAPL1^KO^ and GABARAPL2^KO^ formed a separate, distinct group with ATG7^KO^ (Figure 2), indicating that the three GABARAPs contribute differently to FVIII trafficking, despite their sequence and functional similarities [22,24]. Interestingly, the same divergence in FVIII secretion, associated energy levels, mitochondrial count, and transcriptomic/proteomic analysis was observed in the different GABARAP(s)-KO clones; in this scenario, the GABARAP^KO^ was also closer in phenotypes to CALR^KO^ [18]. Interestingly, further DKO combinations involving GABARAP and the ER chaperones CANX and CALR (CANX^KO^/CALR^KO^, CALR^KO^/GABARAP^KO^ and CANX^KO^/GABARAP^KO^) showed, as well, very similar (almost the same) FVIII intracellular distribution patterns, regardless of their varying FVIII secretion and residual ATP levels (Figure 6 and Figure 7).

Understanding the reasons behind this outcome remains challenging. In fact, it is difficult to identify functional commonalities between GABARAP and the two ER chaperones, given that the discrepancies between the latter two proteins are already not yet fully characterized. CANX and CALR operate in the primary protein quality control system of the ER by recognizing and binding newly synthesized monoglycosylated proteins such as FVIII. This interaction is normally accompanied by a synchronized recruitment of additional ER aiding chaperones like ERp57 [37]. As a result, both function as anchors, slowing down the kinetic flux of glycoproteins and allowing them to reach stable conformations prior to ER release [37,38]. Despite these similarities between the two chaperones, their differential roles in regulating calcium homeostasis, their varying molecular weights, distinct ER sites, as well as CALR’s observed mobility compared to CANX hint to additional divergent functions [37,38,39,40]. For instance, CANX has been described to associate with ER downstream proteasomal disposal pathways [41,42], while, CALR, with its mobility, interacts more dynamically with various substrates and signaling pathways, inside and outside of the ER lumen, but was not reported to be involved in any proteasome-linked processes [14,38,41].

CALR also shows a physical and characteristic connection to GABARAP. Previous research has shown that GABARAP interacts directly with CALR in vitro and it co-localizes (punctuate structure) and co-immunoprecipitates with CALR in the mouse neural precursor and neuroblastoma model N2a (Neuro 2A) cells, as well as in AMO1 multiple myeloma cells, respectively [28,29,30]. Recently, Gulla et al. demonstrated that GABARAP promotes CALR trafficking to the cell surface during Immunogenic Cell Death (ICD), during which, either under stress conditions or cell apoptosis, CALR serves as an “eat-me” signal for phagocytes like dendritic cells [30,42]. However, despite the numerous studies focusing on the interaction between CALR and GABARAP, no clear evidence or explanation on how both proteins cooperate has been defined to date. CALR and GABARAP were not observed to physically complex one another in our cells (Figure S2); however, the effects of their absence on FVIII secretion and intracellular distribution were strikingly very similar. Both GABARAP^KO^ and CALR^KO^ showed nearly identical reductions in FVIII secretion, similar FVIII intracellular distributions, and responded similarly to various chemical treatments (Figure 2, Figure 3 and Figure 6).

CANX has been identified as a co-receptor in ER-phagy, a form of selective autophagy triggered by ER stress (a process which is also induced by FVIII expression and aggregation in the ER) [43,44]. The mechanism by which CANX participates in ER-phagy involves its interaction with the ER-phagy receptor Family with sequence similarity 134, member B (FAM134B) [43]. FAM134B contains a C-terminal LC3-Interacting Region (LIR-domain) which enables its binding to LC3 and GABARAP proteins, thereby promoting the clearance of unwanted or damaged ER components [44]. In the case of certain substrates, such as procollagen I, other components of the ER quality control system, such as CALR, are required for effective elimination to lysosomal degradative pathways [43]. The coordinated interactions between ER chaperones (CANX/CALR), ER-phagy receptors (FAM134B), and autophagy-related proteins (e.g., GABARAP) may therefore explain the similar intracellular profiles of FVIII observed in CANX^KO^, CALR^KO^, and GABARAP^KO^ cells and their respective DKO combinations. This suggests that GABARAP might play an indirect role in ER quality control-associated events, similarly to CANX and CALR, but probably operates downstream in biosynthesis, regulation and homeostatic machineries. While CANX and CALR serve as scaffolds to stabilize misfolded glycoproteins by direct carbohydrate-based binding, linking them to the autophagy machinery via ER-phagy receptors like FAM134B, GABARAP might facilitate the subsequent stages of ER-phagy, influencing therefore the whole homeostatic cellular state. Furthermore, when GABARAP and ER chaperones were “pairwise” deleted, this affected FVIII secretion similar to the single CANX^KO^ and CALR^KO^. This observation suggests two possibilities: either that GABARAP’s role in this context is minimal compared to that of the ER chaperones, or that its effect is masked by the presence and activity of other ER chaperones, given its functional (possibly sequential) link to them.

Furthermore, the unanticipated co-localization of FVIII with markers such as Rab8, VAMP8 and Rab7 (Figure 2), in addition to the effects of BFA, chloroquine and Rab7 inhibition on FVIII secretion (Figure 3), as well as the witnessed positive correlation of FVIII co-localization with endosomal and lysosomal markers (Rab7 and LAMPI) to FVIII secretion levels (Figure 5) in mock conditions, strongly reinforce the presence of FVIII sorting taking place in the *trans*- and post-Golgi networks which may be aiding or happening alongside constitutive FVIII direct exocytosis. For example, upon chloroquine treatment (which inhibits), FVIII secretion correlated less with FVIII presence at *trans*-Golgi markers TGN46 and Rab7, as well as with LC3B at the autophagosomes. On the other hand, under metabolically challenging conditions such as glucose starvation (which also induces autophagy), a remarkable flip in correlation patterns was observed: co-localization of FVIII with trans-Golgi network and subsequent lysosomal markers TGN46, Rab7, and LAMP1 became reduced or negatively correlated, while co-localization with VAMP8 and LC3B became positively or more correlated to FVIII secretion (Figure 5). These findings strongly suggest an active sorting of FVIII within the *trans*-Golgi compartment, with a portion directed toward secretion, yet another potentially toward degradation or recycling, indicating a trifurcation in trafficking pathways. These outcomes also point toward the involvement of multiple possible FVIII secretory routes extending beyond the conventional one, and which could be potentially linked to lysosomal and autophagosomal-related secretions.

Therefore, whether all the FVIII reaching the *trans*-Golgi network is eventually secreted remains an open question. It is also unclear whether FVIII is subject to regulated secretion, whether it is stored in specific vesicles awaiting a trigger, or whether its release is exclusively in a constitutive manner. These possibilities demonstrate that FVIII trafficking and exocytosis are tightly regulated processes, potentially modulated in response to cellular stress (as seen by protein KOs and varying treatment conditions) and to cellular energetic statuses (as evidenced by the correlation of ATP levels to FVIII secretion). Despite these insights, the precise mechanisms and vesicular compartments responsible for efficient FVIII exocytosis, recycling, and intracellular regulation remain largely undefined and demand further investigation.

## 4. Materials and Methods

### 4.1. Generation of CRISPR/Cas9-Knockouts, Cell Culture and Cell Maintenance

The cell lines employed in this study originate from a parental clone of HEK293 cells (designated HEK28) genetically modified to stably express FVIII at a known concentration as described by Al-Rifai et al. [45]. Genetically modified cell lines were generated as described by Singer et al. [18]; using the HEK28^WT^ clone, CRISPR/Cas9 single and double gene knockouts were performed targeting Calnexin, Calreticulin, LMAN1, MCFD2, GABARAP, GABARAPL1, GABARAPL2 and ATG7 proteins. Cells were maintained in Dulbecco’s modified Eagle’s medium (DMEM) supplemented with 10% heat inactivated fetal bovine serum and 1% penicillin-streptomycin mixture. Cells were incubated in a humidified 5% CO_2_ incubator at 37 °C, then passaged, counted and seeded according to downstream experiments.

### 4.2. Genetic Rescue of Double CRISPR/Cas9-Knockouts

HEK28^WT^, single and double CRISPR/Cas9-KO cell lines carrying different knockouts were seeded (250 × 10^3^) in 24-well plates, incubated in a 5% CO_2_ humidified incubator until reaching 70% confluence. Knockout cell lines were then transfected using Lipofectamine 2000 (Thermo Fisher #11668019, Langerwehe, Germany). Lipofectamine was mixed with 2 µg expression plasmids (OriGene, Vector Builder) carrying genes encoding CANX, CALR, LMAN1, MCFD2, and GABARAP (in single and double combinations) in reduced sera-medium OptiMEM (Thermo Fisher #11058021, Langerwehe, Germany) and left to incubate for 20 min at room temperature (RT) before adding the mixture to the cells. Media were collected 48 H following transfection for further FVIII activity assessment.

### 4.3. FVIII Activity Measurement

FVIII: AC assessment ((%) IU/dL) was performed using a two-stage chromogenic substrate assay (CSA) according to the standard protocol established in our laboratory [18,45].

### 4.4. Measurement of Intracellular ATP Concentration

Assessment of intracellular ATP concentration was performed using the Luminescent ATP detection assay kit (#ab113849 Abcam, Cambridge, UK), according to the detailed protocol described by Singer et al. [18].

### 4.5. Western Blot Analysis

Cells were lysed using RIPA buffer supplemented with protease inhibitor cocktail tablets (complete Tablets EDTA-free, EASYpack ROCHE #04693132001, Mannheim, Germany). Protein extracts were mixed with the corresponding ß-mercaptoethanol-containing sample buffer and further denatured at high temperatures (80–95 °C). 2D-SDS/PAGE electrophoresis was performed and proteins were then transferred to PVDF membranes. A day after, membranes were washed extensively with TBS-T (Tris-Buffered Saline with 0.1% Tween-20) and probed with corresponding primary and HRP-conjugated secondary antibodies (Horseradish Peroxidase). Detection was carried out using the ChemiDoc MP Imaging System (Bio-Rad Laboratories GmbH, Feldkirchen, Germany).

### 4.6. Immunofluorescence Staining and Microscopy Imaging

Cells were seeded onto cover slips coated with 0.1% gelatin in 12-well plates in complete DMEM, then incubated until reaching a confluence of 70–80%. Upon incubation, cells were fixed with 4% paraformaldehyde and stored at 4 °C. Indirect labeling of proteins of interest was performed using primary and secondary fluorophore-conjugated secondary antibodies (Table S1). Images were acquired using an Axio Observer ApoTome.2 fluorescence microscope (Carl Zeiss AG, Oberkochen, Germany). Pearson’s Correlation Coefficients were extracted from the Zen Blue 2.6 pro software for the purpose of assessing co-localization values. Protocol information could be precisely found described in [18].

### 4.7. Drug/Chemical and Cell Treatments

All drugs and chemicals were reconstituted at a final specific stock concentration and subsequently diluted in supplemented media.

Glucose Starvation: HEK28^WT^ cells were first subjected to various conditions: (1) reduced serum 5% hiFBS in DMEM #11995-065 Thermo Fisher, (2) medium without glucose: #11966-025 ThermoFischer; (3) medium with low glucose, no glutamine #11054020 Thermo Fisher, and (4) medium without Glucose nor Glutamine: #A1443001 Thermo Fisher for 24 to 36 h. The efficacy of the treatment was verified by Western Blot and Immunofluorescence Staining assays targeting modifications in LC3B and LAMPI proteins, two markers for autophagosomes and lysosomes, respectively (Figure 3A). Glucose deprivation was chosen to treat the selected KO clones simultaneously, starting with the same initial cell count.

Brefeldin A treatment: Brefeldin A was prepared as a 10 mg/mL stock concentration in DMSO and stored −20 °C. HEK28^WT^ cells were treated with decreasing concentrations of Brefeldin A (Biomol GmbH/Cayman Chemical, Item No. 11861, Hamburg, Germany) ranging from 1 µg/mL to 0.1 µg/mL for 6 to 12 h. Treatment efficacy was confirmed through Immunofluorescence Staining targeting modifications in GM130, and TGN46 proteins, well-established markers for the ER, *cis-* and *trans*-Golgi networks, respectively (Figure 3B). An optimal concentration of 0.05 µg/mL was chosen for simultaneous treatment of the selected KO clone, considering the drug’s effectiveness, minimal toxicity level, and the required time frame for FVIII secretion by the cells.

Rab7 inhibition: CID 1,067,700 (SIGMA ALDRICH, SML0545-5MG) was prepared as a 10 mg/mL stock solution in DMSO and stored at −20 °C. Rab7 inhibition was established in HEK28^WT^ with a systematic administration of increasing concentrations of CID 1,067,700 ranging from 5 µM to 40 µM over a duration of 24 to 36 h. Treatment efficacy was confirmed through Western Blot targeting expected modifications in the LAMPI protein, a lysosomal marker (Figure 3C). An optimal concentration of 40 µM was chosen.

Chloroquine Treatment: Chloroquine (CQ) (Cayman Chemical, Item No. 14194) was reconstituted prior to each use at a 50 mM stock concentration in ddH_2_O and then filtered. Increasing concentrations of CQ (ranging from 2.5 µM to 15 µM) were systematically administrated in HEK28^WT^ cells over a duration of 24 to 36 h. The efficacy of treatment was verified by Western Blot and Immunofluorescence Staining assays targeting modifications in the LC3B protein, a recognized autophagosomal marker (Figure 3D). An optimal concentration of 10 µM was chosen, taking into account drug effectiveness and minimal toxicity level, to treat the selected CRISPR/Cas9-KO clones simultaneously, starting with the same initial cell count.

### 4.8. Statistical Analysis and Data Visualization

Statistical analyses were performed using the GraphPad PRISM 6.07 (GraphPad Prism (RRID: SCR_002798) and Word Excel 2016 softwares. Data visualization, including principal component analyses (PCA) plots and Heat Maps matrices, was conducted with Qlucore Omics Explorer 3.6 and with the ClustVis web tool.

## Data Availability

No new patient, human, or animal data were generated or analyzed in this study. Supporting materials related to the reported findings and high resolution raw data are available from the corresponding author upon reasonable request, subject to copyright restrictions and data size limitations.

## Supplementary Materials

The following supporting information can be downloaded at: https://www.mdpi.com/article/10.3390/ijms26136349/s1.

## Abbreviations

The following abbreviations are used in this manuscript:

AC	Activity
AMO1	Human Bone Marrow Plasmacytoma cells
ARL8B	ADP-Ribosylation Factor-Like Protein 8B
ATP	Adenosine Triphosphate
BFA	Brefeldin A
BiP	Binding Immunoglobulin Protein
CALR	Calreticulin
CANX	Calnexin
COPI	Coat Protein Complex I
COPII	Coat Protein Complex II
CQ	Chloroquine
CRISPR	Clustered Regularly Interspaced Short Palindromic Repeats
DKO	Double-KO
DMSO	Dimethyl Sulfoxide
ER	Endoplasmic Reticulum
ERGIC	Endoplasmic Reticulum-Golgi Intermediate Compartment
ERp57	Endoplasmic Reticulum Resident Protein 57
FAM134B	Family with sequence similarity 134, member B
GABARAP	Gamma-Aminobutyric Acid Receptor Associated Protein
GABARAPL1	GABARAP-Like 1
GABARAPL2	GABARAP-Like 2
GAPDH	Glyceraldehyde-3-phosphate dehydrogenase
GM130	Golgi Matrix protein 130
hiFBS	Heat-Inactivated Fetal Bovine Serum
ICD	Immunogenic cell death
KO	Knockout
LAMPI	lysosome-associated membrane protein I
LC3B	Microtubule-associated Protein 1 Light Chain 3 B
LIR	LC3- interacting Region
LMAN1	Lectin Mannose binding 1
LSEC	Liver Sinusoidal Endothelial Cells
MCFD2	Multiple Coagulation Factor Deficiency 2
N2a	Neuro-2a cells
ns	Non significant
PCA	Principal Component Analysis
PCC	Pearson’s Correlation Coefficient
PDI	Protein Disulfide Isomerase
Rab	Ras-like proteins in brain
RIPA	Radioimmunoprecipitation Assay
RT	Room Temperature
S	Sample
SD	Standard Deviation
Sec31a	SEC31 homolog A
SKO	Single-KO
SPR	Surface Plasmon Resonance
TGN46	Trans-Golgi Network Protein 46
Ub	Ubiquitin
V	Variable
VAMP	Vesicle-Associated Membrane Protein
WB	Western Blot
WT	Wild Type
