# Supplementary material for: FVIII Trafficking Dynamics Across Subcellular Organelles Using CRISPR/Cas9 Specific Gene Knockouts
Source: Int J Mol Sci. 2025 Jul 1;26(13):6349. doi: 10.3390/ijms26136349 (PMC12250038; doi:10.3390/ijms26136349)
Supplement: Supplementary file 1 [file ijms-26-06349-s001.zip › ijms-3721741-supplementary.pdf]

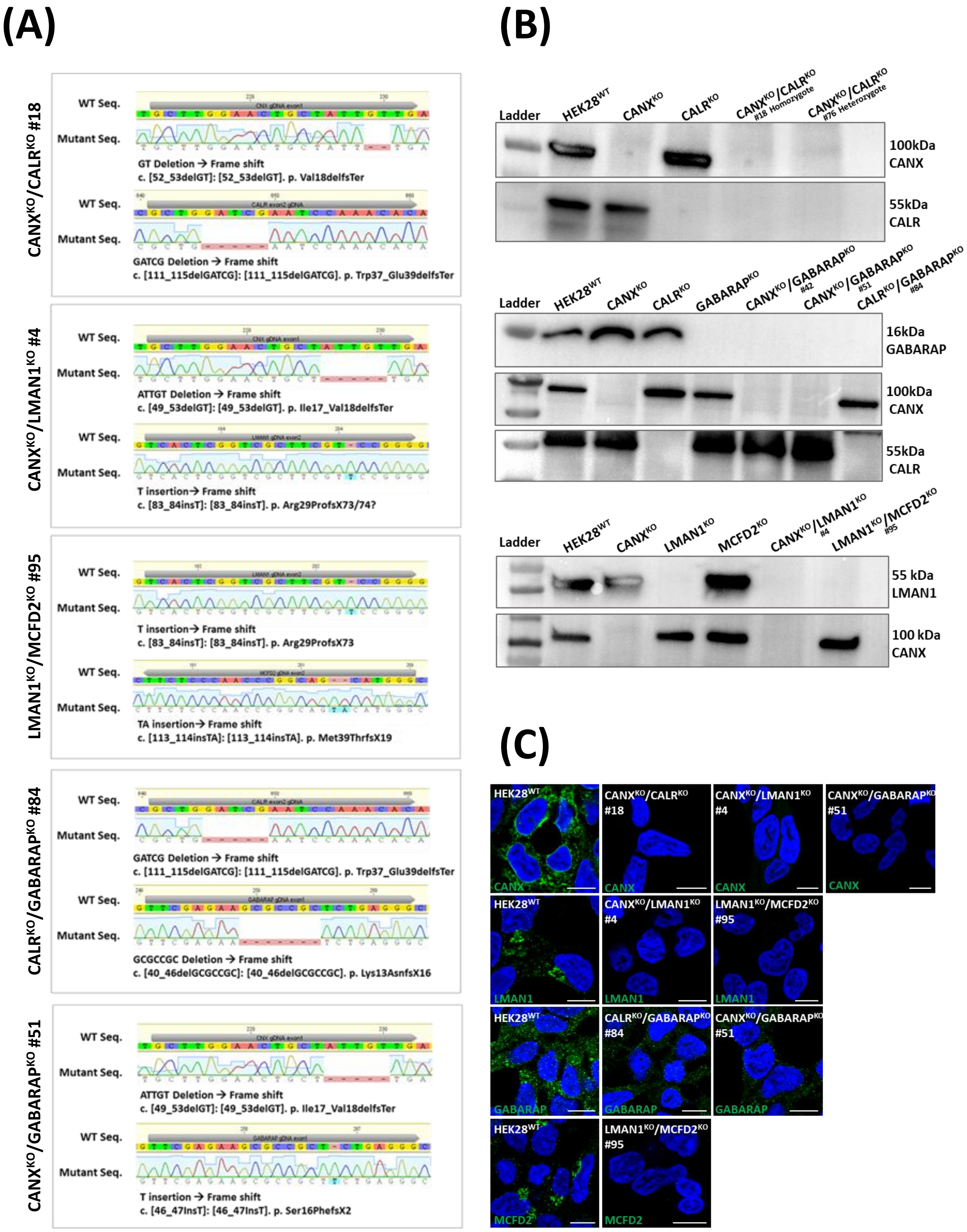

(A)
CANXKO/CALRKO #18
WT Seq.
Mutant Seq.
GT Deletion → Frame shift
c. [52_53delGT]: [52_53delGT]. p. Val18delfsTer
WT Seq.
Mutant Seq.
GATCG Deletion → Frame shift
c. [111_115delGATCG]: [111_115delGATCG]. p. Trp37_Glu39delfsTer
CANXKO/LMAN1KO #4
WT Seq.
Mutant Seq.
ATTGT Deletion → Frame shift
c. [49_53delGT]: [49_53delGT]. p. Ile17_Val18delfsTer
WT Seq.
Mutant Seq.
T insertion → Frame shift
c. [83_84insT]: [83_84insT]. p. Arg29ProfsX73/74?
LMAN1KO/MCFD2KO #95
WT Seq.
Mutant Seq.
T insertion → Frame shift
c. [83_84insT]: [83_84insT]. p. Arg29ProfsX73
WT Seq.
Mutant Seq.
TA insertion → Frame shift
c. [113_114insTA]: [113_114insTA]. p. Met39ThrfsX19
CALRKO/GABARAPKO #84
WT Seq.
Mutant Seq.
GATCG Deletion → Frame shift
c. [111_115delGATCG]: [111_115delGATCG]. p. Trp37_Glu39delfsTer
WT Seq.
Mutant Seq.
GCGCCGC Deletion → Frame shift
c. [40_46delGCGCCGC]: [40_46delGCGCCGC]. p. Lys13AsnfsX16
CANXKO/GABARAPKO #51
WT Seq.
Mutant Seq.
ATTGT Deletion → Frame shift
c. [49_53delGT]: [49_53delGT]. p. Ile17_Val18delfsTer
WT Seq.
Mutant Seq.
T insertion → Frame shift
c. [46_47InsT]: [46_47InsT]. p. Ser16PhefsX2
(B)
Ladder
HEK28WT
CANXKO
CALRKO
CANXKO/CALRKO #18 Homozygote
CANXKO/CALRKO #76 Heterozygote
100kDa CANX
55kDa CALR
Ladder
HEK28WT
CANXKO
CALRKO
GABARAPKO
CANXKO/GABARAPKO #42
CANXKO/GABARAPKO #51
CALRKO/GABARAPKO #84
16kDa GABARAP
100kDa CANX
55kDa CALR
Ladder
HEK28WT
CANXKO
LMAN1KO
MCFD2KO
CANXKO/LMAN1KO #4
LMAN1KO/MCFD2KO #95
55 kDa LMAN1
100 kDa CANX
(C)
HEK28WT
CANXKO/CALRKO #18
CANXKO/LMAN1KO #4
CANXKO/GABARAPKO #51
CANX
HEK28WT
CANXKO/LMAN1KO #4
LMAN1KO/MCFD2KO #95
LMAN1
HEK28WT
CALRKO/GABARAPKO #84
CANXKO/GABARAPKO #51
GABARAP
HEK28WT
LMAN1KO/MCFD2KO #95
MCFD2

Figure S1. Validation of CRISPR/Cas9-Mediated Double Knockouts. (A) Representative DNA sequence chromatograms from Geneious software confirming the presence and type of mutations, along with corresponding nomenclature, for each double knockout (DKO) combination, compared to the WT un-mutated clone. (B) Western blot analysis of cell lysates demonstrating the complete loss of target proteins in knockout cells, validating successful gene disruption, with WT clones serving as controls. (C) Immunofluorescence (IF) - staining of 4% paraformaldehyde-fixed cells, further illustrating protein loss. Proteins of interest are visualized in green, while nuclei are stained with DAPI (blue). Images were acquired using the ApoTome.2 by Carl Zeiss microscope at 40x magnification with an oil-immersion objective (numerical aperture 1.4), providing an optical resolution of approximately 240 nm in x and y-axes. A 702 monochrome camera with a 6.5 μm pixel pitch was used, yielding a pixel-based resolution of approximately 147 nm. Scale bar: 10 μm.

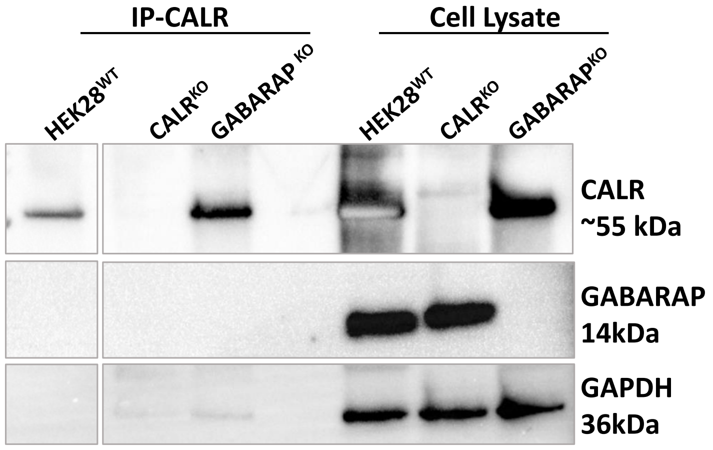

Figure S2. Assessment of Calreticulin–GABARAP Physical Interaction by Immunoprecipitation (IP) and Co-Immunoprecipitation (Co-IP) Assays. Western blot analysis of protein extracts from HEK28^{WT}, CALR^{KO}, and GABARAP^{KO} cells following immunoprecipitation (IP) of CALR. Cell lysis was performed using NP-40 buffer. Proteins were separated by SDS-PAGE using 16% Novex™ Tris-Tricine Mini Protein Gels (*Thermo Fisher, Langerwehe, Germany*) for 45 min at 220 V and transferred onto a PVDF membrane for 1 hour at 250 mA. The PVDF-membrane was probed with primary antibodies against CALR, GABARAP, and GAPDH, followed by appropriate HRP-conjugated secondary antibodies. Details of the antibodies used are provided in Table S1.

Antibody	Supplier/Reference	Use	Additional Identifier
FVIII	Affinity Biologicals	IF	SAF8C-AP/ AP1871-BR1
PDI	Cell Signaling Technology Cat# 3501, RRID:AB_2156433	IF	C81H6
Calreticulin	Santa Cruz Biotechnology Cat# sc-11398, RRID:AB_667959	IF	Calregulin (H-170)
Calreticulin	Abcam Cat# ab22683, RRID:AB_447253	WB	ab22683
Calreticulin	Novus Biologicals #NB600-103	IP	Calreticulin Antibody - BSA Free ##NB600-103
Calnexin	Santa Cruz Biotechnology Cat#sc-46669	IF/WB	Calnexin (E-10)
LMAN1 (ERGIC-53)	Santa Cruz Biotechnology Cat# sc-271517, RRID:AB_10649805	IF/WB	ERGIC-53 (B-9)
MCFD2	Santa Cruz Cat# sc-390463	IF	MCFD2 (F-3)
Sec31a (COPII)	Cell signaling Technology Cat# 13466, RRID:AB_2798228	IF	D1G7I
COPB (COPI)	Santa Cruz Cat# sc-393615	IF	COPB (D-10)
GM130	Cell Signaling Technology Cat# 12480, RRID:AB_2797933	IF	D6B1
TGN46	Sigma-Aldrich Cat# T7576, RRID:AB_1080261	IF	T576
Furin	Abcam Cat# ab3467, RRID:AB_303828	IF	3467
Rab5	Cell Signaling Technology Cat# 3547, RRID:AB_2300649	IF	C8B1
Rab7	Santa Cruz Biotechnology Cat# sc-376362, RRID:AB_10987863	IF	Rab7 (B-3) sc-376362
Rab8	Cell Signaling Technology Cat# 6975, RRID:AB_10827742	IF	D22D8

Rab11	Cell Signaling Technology Cat# 5589, RRID:AB_10693925	IF	D4F5
Rab26	Proteintech Cat# 14284-1-AP, RRID:AB_2176709	IF	14284-1-AP
LAMPI	Cell Signaling Technology	IF/WB	D2D11
ARL8B	Proteintech Cat# 13049-1-AP, RRID:AB_2059000	IF	13049-1-AP
VAMP8	Santa Cruz Biotechnology Cat# sc-166820, RRID:AB_2212959	IF	Endobrevin (G-12) sc- 166820
LC3B	Abcam Cat# ab51520, RRID:AB_881429	IF/WB	51520
GABARAP	R and D Systems Cat# MAB8574, RRID:AB_3659211	IF	MAB8574/ Clone#853641
GABARAP	Cell Signaling Technology Cat# 13733, RRID:AB_2798306	WB	E1J4E
GABARAPL1	Proteintech Cat# 11010-1-AP, RRID:AB_2294415	IF	11010-1-AP
GAPDH-HRP	(Santa Cruz Biotechnology Cat# sc-47724, RRID:AB_627678)	WB	sc-47724
Ubiquitin	Santa Cruz	IF	Ubiquitin (F-11) sc-271289
Donkey anti-Sheep Alexa Fluor 594	ThermoFisher scientific	IF	A- 11016
Goat anti-Mouse Alexa Fluor 488	ThermoFisher scientific	IF	A-11029
Donkey anti-Rabbit Alexa Fluor 488	ThermoFisher scientific	IF	A11008
Donkey anti-Rat Alexa Fluor 488	ThermoFisher scientific	IF	A-21208
Donkey anti-Mouse Alexa Fluor 488	ThermoFisher scientific	IF	A-21202
Anti-Rabbit HRP	Abcam Cat# ab6721, RRID:AB_955447	WB	6721
Anti-Mouse HRP	Abcam Cat# ab6789, RRID:AB_955439	WB	6789

Table S1. Table representing the information on the set of antibodies employed in this study
